# Impact of Qi Gong therapy for managing premenstrual syndrome among adolescent girls

**DOI:** 10.6026/973206300210132

**Published:** 2025-02-28

**Authors:** Vaghela Payalben Tejmalji, Mahalakshmi B., Siva Subramanian N.

**Affiliations:** 1 Department of Obstetrics and Gynecological Nursing, Nootan college of Nursing, Sankalchand Patel university, Visnagar, Gujarat - 384315, India; 2 Department of Pediatric Nursing, Nootan College of Nursing, Sankalchand Patel University, Visnagar, Gujarat - 384315, India; 3 Department of Psychiatric Nursing, Nootan College of Nursing, Sankalchand Patel University, Visnagar, Gujarat - 384315, India

**Keywords:** Premenstrual syndrome, Qi gong therapy, adolescent girls, non-pharmacological interventions, holistic health

## Abstract

Premenstrual Syndrome (PMS) is a prevalent condition among adolescent girls, causing significant physical and emotional distress. Therefore, it is of interest to evaluate the impact of Qi Gong therapy on alleviating PMS symptoms among adolescent girls in North Gujarat. A quasi-experimental pretest-posttest design was implemented with 231 adolescent girls aged 13-17 years. Participants underwent a 4-week Qi Gong therapy program, with five 45-minute sessions weekly. Data were collected using a demographic questionnaire and Modified PMS Scale, analysing pre - and post-intervention symptoms through descriptive statistics, paired t-tests and chi-square tests. The intervention significantly reduced PMS severity, with mild PMS cases increasing from 48 (20.78%) to 166 (71.86%) post-intervention. Paired t-tests revealed a highly significant mean difference in PMS scores (T = 12.251, p < 0.001).

## Background:

Premenstrual Syndrome (PMS) is a complex cyclical disorder affecting 30-50% of reproductive-aged women, with significant prevalence among adolescent girls [1]. Characterized by physical, emotional and behavioural symptoms occurring during the luteal phase, PMS can substantially impair daily functioning and quality of life [2]. Conventional PMS management strategies typically involve pharmacological interventions, which often present limitations including side effects and incomplete symptom resolution [3]. Consequently, there is growing interest in non-pharmacological, holistic approaches that address symptom complexity while minimizing potential adverse reactions. Qi Gong, a traditional Chinese mind-body practice combining movement, breathing techniques and meditation, represents a promising alternative intervention. Emerging research suggests potential therapeutic benefits in managing hormonal and psychological symptoms associated with reproductive health [4].

Despite increasing recognition of PMS's impact, adolescent populations remain underexplored in comprehensive intervention studies. Most existing research focuses on adult populations, creating a critical knowledge gap in understanding age-specific management strategies [5]. Therefore, it is of interest to systematically evaluate Qi Gong therapy's effectiveness in alleviating PMS symptoms among adolescent girls, addressing a significant research deficit and exploring innovative non-pharmacological intervention strategies.

## Methodology:

## Research design and setting:

Quasi-experimental, pretest-posttest design was conducted [6, 7] in selected schools in North Gujarat.

## Population and sampling target population:

Adolescent girls aged 13-17 years experiencing PMS Sample Size: 231 participants.

## Sampling method:

Stratified random sampling

## Intervention:

The intervention consists of a supervised Qi Gong therapy program, conducted 5 sessions per week for 4 weeks, with each session lasting 45 minutes and focusing on slow movements, controlled breathing and meditation.

## Data collection tools & data analysis:

Data collection involves a demographic questionnaire and a modified PMS scale. Ethical considerations include obtaining informed consent, securing institutional ethics committee approval and ensuring participant confidentiality. Data will be analyzed using descriptive statistics, paired t-tests and chi-square tests, with a significance level set at p < 0.05.

## Results:

Table 1 shows that largest age group being above 15 years (28.56%), nearly equal dietary preferences with vegetarians at 50.22% and non-vegetarians at 49.78% and a majority attaining menarche after 12 years (60.17%). Physical exercise was reported by 44.59% of participants, while 55.41% did not exercise. Notably, most participants experienced menstrual flow for 5-7 days (36.8%) and used 4-5 pads daily (46.75%). Table 2 reveals the statistical significance of the reduction in PMS scores post-intervention. The paired t-test showed a mean difference of 23.233 with a t-value of 12.251 and a highly significant p-value (< 0.001), confirming the effectiveness of Qi Gong therapy in alleviating PMS symptoms among adolescent girls. Table 3 illustrates associations between PMS severity and socio-demographic variables. Significant associations were observed with age (p = 0.014), physical exercise (p = 0.038), frequency of menstrual cycle (p = 0.037), days of menstrual flow (p = 0.001), family history of PMS (p = 0.004) and days experiencing PMS (p = 0.001). These findings highlight the influence of lifestyle and biological factors on PMS severity. Figure 1 (see PDF) shows a marked improvement in PMS scores following the intervention. The mean pretest score of 86.455 dropped to 63.221 in the posttest, with a mean difference of 23.233. The standard deviation also reduced from 26.073 (pretest) to 24.402 (posttest), indicating less variability in post-intervention symptoms.

## Discussion:

Premenstrual Syndrome (PMS) is a multifaceted condition affecting women's physical, emotional and behavioural well-being, often impairing daily functioning. Non-pharmacological and complementary therapies, such as Qi Gong, have shown promise as holistic approaches to PMS management. Qi Gong, a traditional mind-body practice, focuses on controlled breathing, movement and meditation to restore balance in the body. Recent studies highlight its ability to alleviate PMS symptoms effectively, offering a sustainable alternative to pharmacological treatments. The efficacy of Qi Gong in reducing PMS symptoms has been supported by multiple studies. Jang *et al.* [8] demonstrated that Qi Gong significantly reduced pain, depression and anxiety among women with PMS by restoring Qi balance. Similarly, Armour *et al.* [9] found that Qi-based therapies, including acupuncture and acupressure, improved both physical and emotional symptoms of PMS. Lee *et al.* [10] reported that Qi Gong effectively reduced pain and tenderness in fibromyalgia patients, suggesting broader applicability in stress-related conditions like PMS. Compared to other complementary therapies, Qi Gong demonstrates unique benefits. Viswanathan *et al.* [11] found that meditation-based interventions, such as music-based chakra meditation, reduced PMS symptoms by promoting relaxation and hormonal balance. Furthermore, studies like Ryu and Kim [12] emphasize the importance of complementary approaches in managing PMS, noting that while pharmacological treatments such as antidepressants can be effective, therapies like Qi Gong provide long-term symptom relief with minimal side effects. Xu *et al.* [13] also highlighted the role of herbal treatments targeting liver-qi stagnation, which can work synergistically with Qi Gong to improve emotional well-being.

The mechanisms underlying Qi Gong's effectiveness are rooted in its ability to regulate the hypothalamic-pituitary-adrenal (HPA) axis, lower cortisol levels and enhance parasympathetic activity. These physiological changes help balance hormones, alleviate stress and improve overall mental health, as noted by Ulbricht *et al.* [14]. Additionally, Bongi *et al.* [10] found that Qi Gong's integration with physical therapies reduced psychological distress and enhanced hormonal homeostasis, further validating its role in PMS management. Beyond physical symptom relief, Qi Gong addresses emotional disturbances often associated with PMS. Studies like Siminiuc and Turcanu [15] suggest that dietary interventions, when combined with Qi Gong, can enhance mood and emotional stability. Similarly, Sun *et al.* [16] highlighted the benefits of traditional Chinese Medicine formulas like Baixiangdan Capsules in regulating liver-qi and complementing the emotional benefits of Qi Gong. Our study aligns with previous research demonstrating the effectiveness of Qi Gong therapy in alleviating PMS symptoms. Jang and Lee (2004) found significant improvements in negative emotions, pain and water retention among women receiving Qi therapy [17] Jang *et al.* (2004) further reported reductions in pain, depression and anxiety, highlighting its role in restoring Qi balance [18]. Additionally, Chen *et al.* (2006) found benefits in managing pain-related conditions. Consistent with these findings, our study confirms that Qi Gong therapy is a promising non-pharmacological approach to reducing PMS symptoms in adolescent girls [19]. Van Dam [20] further highlighted the interdisciplinary approach of Qi Gong in addressing workplace stress, which often exacerbates PMS symptoms.

## Conclusion:

Qi Gong therapy offers a holistic, non-invasive approach for managing PMS by addressing both physiological and emotional dimensions to the condition. Its ability to balance hormones, alleviate stress and improve overall quality of life makes it a valuable addition to PMS care.

## Figures and Tables

**Table 1:** Demographic characteristics of participants (QI gong therapy group)

**S.No.**	**Variable**	**Category**	**Frequency (n)**	**Percentage (%)**
1	Age	13 years	60	25.97
		14 years	61	26.42
		15 years	44	19.05
		Above 15 years	66	28.56
2	Religion	Hindu	113	48.92
		Muslim	59	25.54
		Christian	35	15.15
		Others	24	10.39
3	Diet	Vegetarian	116	50.22
		Non-Vegetarian	115	49.78
4	Weight (kg)	Less than 31 kg	13	5.63
		31-35 kg	59	25.54
		36-40 kg	52	22.51
		41-45 kg	51	22.08
		46-50 kg	51	22.08
		Above 50 kg	5	2.16
5	Birth Order	First Child	69	29.87
		Second Child	91	39.39
		Third Child and Above	71	30.74
6	Type of Beverage Consumed	Coffee	36	15.58
		Tea	47	20.35
		Milk	50	21.65
		Fruit Juice	45	19.48
		None	53	22.94
7	Physical Exercise	Yes	103	44.59
		No	128	55.41
8	Hours of Sleep Per Day	< 5 hours	7	3.03
		5-7 hours	113	48.92
		8-10 hours	111	48.05
		> 10 hours	0	0
9	Age at Menarche	< 12 years	92	39.83
		> 12 years	139	60.17
10	Frequency of Menstrual Cycle	26-28 days	55	23.81
		29-31 days	63	27.27
		32-34 days	50	21.65
		Above 35 days	63	27.27
11	Duration of Menstrual Flow	2-4 days	82	35.5
		5-7 days	85	36.8
		8-10 days	64	27.7
12	Pads Changed Per Day	Less than 4 pads	88	38.1
		4-5 pads	108	46.75
		More than 5 pads	35	15.15
13	Family History of PMS	Present	54	23.38
		Absent	177	76.62
14	Days Experiencing PMS	1-3 days	74	32.03
		4-6 days	100	43.29
		7-10 days	53	22.94
		Above 10 days	4	1.74

**Table 2:** Paired t-test comparison of pretest and post-test PMS scores in QI gong therapy group (n = 231)

**Group**	**Test**	**Mean**	**S.D**	**Mean Difference**	**t-test Value**	**p-Value**	**Result**
Qi Gong Therapy	Pretest	86.455	26.073	23.233	12.251	< 0.001	Significant
	Posttest	63.221	24.402				

**Table 3:** Chi-square test for association between pretest PMS scores and socio-demographic variables (n = 231)

**S.N.**	**Demographic variable**	**Chi-Square value**	**Degrees of freedom (DF)**	**p-value**	**Result**
1	Age	20.657	9	0.014	Significant
2	Religion	8.981	9	0.439	Not Significant
3	Diet	2.679	3	0.444	Not Significant
4	Weight (kg)	9.018	15	0.877	Not Significant
5	Birth Order	7.908	6	0.245	Not Significant
6	Type of Beverage	9.141	12	0.691	Not Significant
7	Physical Exercise	8.4	3	0.038	Significant
8	Hours of Sleep Per Day	1.05	6	0.984	Not Significant
9	Age at Menarche	1.887	3	0.596	Not Significant
10	Frequency of Menstrual Cycle	17.854	9	0.037	Significant
11	Days of Menstrual Flow	24.025	6	0.001	Significant
12	Pads Changed Per Day	8.58	6	0.199	Not Significant
13	Family History of PMS	13.241	3	0.004	Significant
14	Days Experiencing PMS	33.345	9	0.001	Significant
